# Jinghuaweikang capsule alleviates *Helicobacter pylori*-infected gastric mucosal inflammation and drug resistance by regulating intestinal microbiota and MAPK pathway

**DOI:** 10.3389/fcimb.2025.1628594

**Published:** 2025-11-28

**Authors:** Yao Yang, Xiao-Fen Jia, Guang-Hui Cui, Qiu-Yue Huang, Miao-Miao Lin, Zong-Ming Shi, Hui Ye, Xue-Zhi Zhang

**Affiliations:** 1 Department of Integrated Traditional Chinese and Western Medicine, Peking University First Hospital, Beijing, China; 2 Institute of Integrated Traditional Chinese and Western Medicine, Peking University, Beijing, China

**Keywords:** *Helicobacter pylori* infection, inflammation, drug resistance genes, intestinal microbiota, metagenomic sequencing

## Abstract

**Background:**

*Helicobacter pylori (H. pylori)* infection represents a prevalent global health burden. Current eradication strategies are complicated by increasing antibiotic resistance and detrimental alterations to the gut microbiome. Jinghuaweikang capsule (JWC), a traditional Chinese medicine, has demonstrated efficacy against *H. pylori*, yet its mechanisms involving microbiota-inflammation interactions remain incompletely elucidated.

**AIM:**

This study aimed to investigate the effects of the JWC on gastric mucosal inflammation and the expression of drug-resistance genes in *H. pylori*-infected mice.

**Methods:**

Sixty Kunming mice were randomly allocated into six groups, including normal control group (Control), model group (Model), Western medicine triple group (AC), low-dose JWC group (JWCL), medium-dose JWC group (JWCM), and high-dose JWC group (JWCH). A mouse model of *H. pylori* infection was established by intragastric administration of an *H. pylori* SS1 solution for two weeks. The efficacy of this model was evaluated using rapid urease test (RUT) and Warthin-Starry (WS) silver stain. Subsequently, the experimental cohort of mice underwent pharmacological intervention. Hematoxylin and eosin (HE) staining, enzyme-linked immunosorbent assay (ELISA), and quantitative real-time polymerase chain reaction (qRT-PCR) were used to assess the impact of JWC on inflammation within the gastric mucosa of mice infected with *H. pylori*. Metagenomic sequencing technology was used to identify alterations in the intestinal microbiota and antibiotic resistance genes in the murine models. Western blotting was used to assess the expression levels of proteins involved in the mitogen-activated protein kinase (MAPK) signaling pathway.

**Results:**

JWC mitigated gastric mucosal inflammation induced by *H. pylori* infection and reduced the concentrations of interleukin- (IL-) 6, IL-1β, and tumor necrosis factor-α (TNF-α) while inhibiting gene expression level. Metagenomic sequencing revealed that triple therapy in Western medicine markedly diminished the diversity of the intestinal microbiota while elevating the abundance of antibiotic-resistance genes, including *macB*, *arlR*, *evgS*, *tetA(58)*, and *mtrA*. The diversity and richness of the intestinal microbiota in the JWC group were comparable to those in the control group, with an increase in the abundance of beneficial bacteria such as *Muribaculaceae_bacterium*. Furthermore, the expression levels of the antibiotic resistance genes *macB*, *tetA(58)*, *bcrA*, *oleC*, *and arlS* were downregulated. Moreover, the activation of MAPK signaling pathway components phospho-ERK and phospho-p38 was inhibited.

**Conclusion:**

JWC preserves microbial diversity and promotes a beneficial compositional shift, mitigates the risk of antibiotic resistance, modulates the MAPK signaling pathway, and alleviates gastric mucosal inflammation in mice infected with *H. pylori*.

## Introduction

*Helicobacter pylori* (*H. pylori*), a prominent gut microbe, is among the most extensively studied bacteria. The global prevalence of *H. pylori* infection is estimated to be 48.5%, and approximately 55.8% of the population in China is affected by this infection (Hooi et al., 2017). Eradicating *H. pylori* can mitigate gastric mucosal inflammation and decrease the risk of developing gastric cancer (Helicobacter pylori Group CSoG and Zhou, 2022). The widespread implementation of triple or quadruple therapies, comprising proton pump inhibitors (PPIs) and antibiotics, as the primary strategy for eradicating *H. pylori* has resulted in the emergence of antibiotic resistance and disruption of the intestinal microbiota (Liou et al., 2019; Liou et al., 2020). Recently, the global prevalence of *H. pylori* antibiotic resistance has escalated to a concerning level (Herzlinger et al., 2023; Wang Y. et al., 2023; Hong et al., 2024; Okimoto et al., 2024; Xu et al., 2024). Moreover, gut microbiota dysbiosis has been increasingly recognized as a key contributor to the persistence of *H. pylori* infection and the associated inflammatory response. Furthermore, disruption of the intestinal microbiota is associated with an increased risk of various human diseases, including inflammatory bowel disease (Nguyen et al., 2020), obesity (Cox and Blaser, 2015), colorectal cancer (Akimoto et al., 2021), and gastrointestinal infections (Johnson et al., 1999; Lynch and Martinez, 2002). The duration and dosage of antibiotics can no longer be increased further, and the range of available antibiotics is severely restricted. The search for new paths to eradicate *H. pylori* is an inevitable path for treating *H. pylori*.

In contrast to conventional antibiotic-based regimens, traditional Chinese medicine offers a holistic approach that may restore microbial diversity and mitigate inflammation with fewer adverse effects on commensal flora (Qu et al., 2022; Gong et al., 2024; Wang et al., 2024). However, the precise mechanisms underlying these beneficial effects remain poorly understood, necessitating further investigation. Jinghuaweikang capsule (JWC) is the most frequently utilized traditional Chinese medicine for the clinical management of *H. pylori*, formulated from potent ingredients derived from *chenopodium ambrosioides* and *adina pilulifera*. Additionally, JWC is the sole traditional Chinese medicine explicitly endorsed by the guidelines for managing refractory *H. pylori* infections (Helicobacter pylori Study Group, 2022). Previous *in vitro* research has demonstrated that JWC and chenopodium oil can inhibit and eradicate standard and resistant *H. pylori* strains. Furthermore, JWC has been demonstrated to reduce the expression of the hefABC active efflux pump system in *H. pylori* (Liu, 2014), and chenopodium oil effectively inhibits the formation of biofilms associated with resistant *H. pylori* (Zhang et al., 2020). In addition, the combination of JWC with bismuth therapy in triple or quadruple regimens has been exhibited to enhance the eradication rate of *H. pylori*, alleviate clinical symptoms, and decrease the incidence of adverse events in patients with *H. pylori*-related gastritis (Wang et al., 2013; Zhang et al., 2013). Despite these promising findings, *in vivo* studies on the mechanism of JWC against *H. pylori* remain scarce. In this study, we investigated the impact of JWC on the intestinal microbiota and antibiotic-resistance genes through metagenomic sequencing. Moreover, we evaluated the effect of JWC on gastric mucosal inflammation and examined the changes in MAPK pathway proteins in *H. pylori*-infected mice.

## Materials and methods

### Culture and collection of *H. pylori* strains

The standard *H. pylori* strain SS1, which is positive for both the cytotoxin-associated gene A (CagA) and the vacuolating cytotoxin A (VacA), generously provided by the Department of Gastroenterology at Peking University First Hospital, was preserved at −80°C in a low-temperature freezer. The cryopreservation solution was formulated using Brain Heart Infusion (OXOID, Basingstoke, UK) in conjunction with glycerol (Solarbio, Beijing, China). The bacterial suspension was inoculated onto Columbia blood agar plates (OXOID, Basingstoke, UK) enriched with 8% sheep blood (LABLEAD, Beijing, China) and incubated under microaerophilic conditions (85% N_2_, 10% CO_2_, and 5% O_2_) at 37°C for 48–72 h (Lin et al., 2024). Subsequently, the positive colonies were subcultured. The bacteria were collected in Brucella broth (BD, Franklin Lakes, NJ, USA) before intragastric administration.

### Preparation of intervention drugs

The volatile oil of JWC (Tianshi Li, Tianjin, China) exhibited a density of 937 mg/mL, was dissolved in edible oil, and then administered by gavage. The pharmaceutical powders of amoxicillin (Aladdin, Shanghai, China), clarithromycin (Aladdin, Shanghai, China), and lansoprazole (Aladdin, Shanghai, China) were solubilized in double-distilled water and administered by gavage. The representative gas chromatographic fingerprint of JWC is provided in **Supplementary Material Figure S1.**

### Grouping and handling experimental animals

Sixty specific pathogen-free male Kunming (KM) mice (Beijing Vital River Laboratory Animal Technology Co., Ltd.) weighing between 18 and 22 g were kept in a barrier environment at the Experimental Animal Center of Peking University First Hospital (license number: SCXK (Jing) 2024-0005). The mice were adaptively housed for three days, during which they had unrestricted access to water and food.

The mice in the other groups were administered cyclophosphamide (200 mg/kg) via intraperitoneal injection to transiently suppress the immune system and facilitate the subsequent colonization of *H. pylori* (Ye et al., 2015), followed by intragastric administration of 0.3 mL *H. pylori* SS1 solution (12×10^8^ CFU/mL) every other day for seven doses. The mice in the Control group received an equivalent volume of normal saline through intraperitoneal injection and intragastric administration. Two weeks following the final gavage, one mouse from each group was randomly sacrificed to assess *H. pylori* colonization using rapid urease test (RUT) and Warthin-Starry (WS) silver stain.

**Figure 1:**
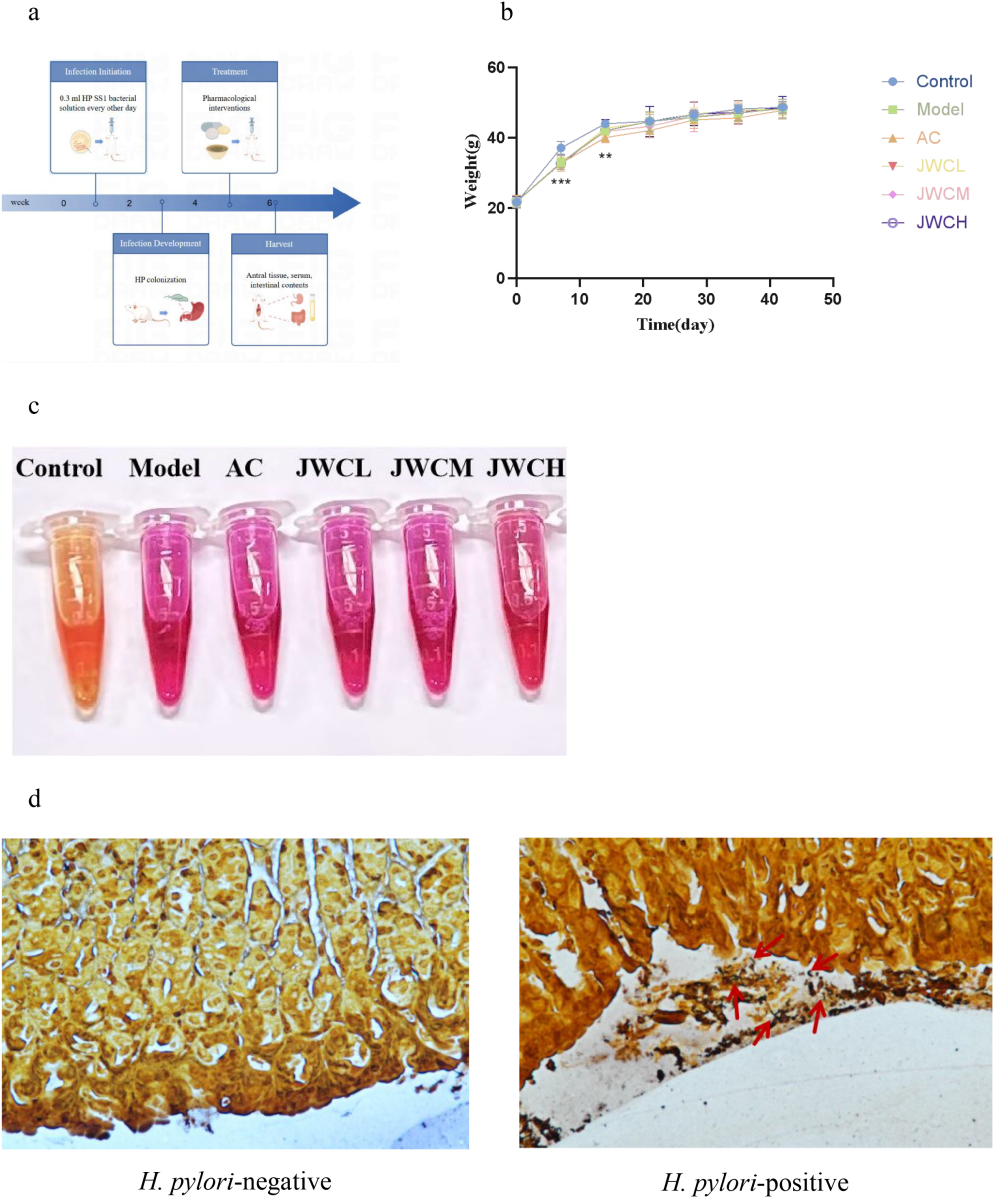
Animal treatment and model determination. **(a)** Flow chart of modeling and drug intervention. **(b)** Changes in body weight of mice during modeling and drug intervention. **(c)** RUT. The solution turning red indicated the presence of *H*. *pylori* infection. **(d)** WS. The red arrow indicated *H*. *pylori* colonized in the gastric mucosa.

Following the successful establishment of the model, the drug intervention was administered the following day. The dosages used for the animal experiments are detailed in **Table 1**. For an adult weighing 60 kg, the conversion formula was Db = Da × Rab, where the conversion coefficient Rab was 12.33. The JWCH group received JWC at the clinical equivalent dose (98.64 mg/kg), while the JWCL and JWCM groups were administered JWC at one-quarter (24.66 mg/kg) and one-half (49.32 mg/kg) of this dose, respectively. Control and Model mice received equal volumes of physiological saline via gastric lavage. The mice were euthanized 14 days after oral administration, antral tissues were harvested, serum was separated, and intestinal contents were collected (**Figure 1a**). The body weight and behavioral patterns of the mice were monitored weekly during the gavage procedure. The experimental protocol was approved by the Experimental Animal Ethics Committee of Peking University First Hospital (approval number: J202122).

**Table 1:** Animal experimental dosage.

Drug	Clinical dosage	Animal experimental dosage
JWC	480mg/d	98.64mg/kg
amoxicillin	2000mg/d	411mg/kg
clarithromycin	1000mg/d	205.5mg/kg
lansoprazole	60mg/d	12.33mg/kg

### Rapid urease test

The entire stomach of each mouse was isolated, an incision was made along the greater curvature, the gastric contents were thoroughly rinsed with distilled water, and a portion of the gastric antral tissue was subsequently transferred into an Eppendorf tube containing rapid urease solution. Any color changes were monitored in the solution, and these observations were documented using photographs. It was necessary to focus on the sampling site and ensure that the tissue size was consistent in each group. The sampling tools were sterilized to avoid cross-contamination.

### Warthin–Starry silver stain

The WS silver stain kit (Beijing Solarbio Technology Co. LTD. Beijing, China) was used for the subsequent experiment. The paraffin sections of mouse gastric antral tissue sections were deparaffinized in water and then stained in acidic Ag solution in a water bath at 56°C for 1 h. The sections were immersed in the prepared staining solution (B1: B2: B3 = 3:9:4) and placed in a water bath at 56°C. They were stained until a yellow-brown color was achieved, subsequently removed, and washed with preheated distilled water at 56°C. The sections were removed until they turned yellowish-brown color and then rinsed with preheated distilled water at a temperature of 56°C. The sections were dehydrated in absolute ethanol, clarified in xylene, and sealed with neutral gum. Finally, the sections were photographed and analyzed under a microscope. The precautions for tissue sampling were the same as before.

### Hematoxylin and eosin staining

The antrum tissue sections of the mice were deparaffinized and hydrated, followed by staining the nucleus with hematoxylin and the cytoplasm with eosin. Subsequently, dehydration was performed again, followed by xylene transparency. Finally, the slices were sealed with neutral gum to fix them and prevent deformation.

### Measurement of IL-6, IL-1β, TNF-α in supernatants of mouse gastric antral tissue

Enzyme-linked immunosorbent assay (ELISA) kits for IL-6 (RK00008, ABclonal), IL-1β (RK00006, ABclonal), and TNF-α (RK00027, ABclonal) were used to detect the levels of inflammatory factors in the supernatants of the gastric antral tissue.

### Quantitative real-time polymerase chain reaction

RNA was extracted from the gastric antral tissue of mice using TRIzol reagent. Following the determination of the RNA concentration, complementary DNA (cDNA) was synthesized using a cDNA Synthesis Kit (Takara, Japan). The relative expression levels of mRNA were quantified employing the 2^−△△Ct^ method (Chu et al., 2022). The primer sequences utilized for quantitative reverse transcription PCR (qRT-PCR) are presented in **Table 2**. β-Actin served as the reference gene.

**Table 2:** Primer sequences.

Primer name	Sequence (5’→3’)	Source
β-Actin fwd	GTGACGTTGACATCCGTAAAGA	This study
β-Actin rev	GCCGGACTCATCGTACTCC	This study
IL-1β fwd	CTGTGACTCATGGGATGATGATG	This study
IL-1β rev	CGGAGCCTGTAGTGCAGTTG	This study
IL-6 fwd	CTGCAAGAGACTTCCATCCAG	This study
IL-6 rev	AGTGGTATAGACAGGTCTGTTGG	This study
TNF-α fwd	CAGGCGGTGCCTATGTCTC	This study
TNF-α rev	CGATCACCCCGAAGTTCAGTAG	This study

### Western blotting

Protein concentration was assayed in RIPA lysis buffer containing protease inhibitors and PMSF using a bicinchoninic acid kit (Beyotime Biotechnology, Shanghai, China). Electrophoresis was performed using 4%–12% precast gels (LABLEAD, Beijing, China) and transferred to PVDF membranes (Millipore, USA). The membranes were blocked in 5% BSA for 1 h at room temperature and then stored overnight at 4°C containing the corresponding antibodies. After washing the membranes with TBST, the secondary antibodies were left at room temperature for 1 h. Images of the membranes were obtained using a BIO-RAD instrument (BIO-RAD Laboratories, Hercules, USA). The following antibodies were used: β-Actin (4970S; 1:1000; CST), anti-p38 (8690T; 1:1000; CST), anti-p-p38 (4511T; 1:1000; CST), anti-ERK (4695T; 1:1000; CST), and anti-p-ERK (4370T; 1:1000; CST).

### Macrogenome sequencing

The purity and integrity of the extracted DNA samples were assessed using agarose gel electrophoresis. The DNA was subsequently fragmented to obtain a fragment size of approximately 350 bp using Covaris M220 (Gene Corporation, China). PE libraries were constructed using end repair, adaptor addition, and PCR. Library sequencing was performed on the Illumina NovaSeq™ X Plus platform (Illumina, USA). The sequence assembly was optimized using MEGAHIT software (https://github.com/voutcn/megahit, version 1.1.2). Gene prediction was conducted using Prodigal software (https://github.com/hyattpd/Prodigal, version 2.6.3), and gene indexes, along with their abundances, were obtained. Diamond software (https://github.com/bbuchfink/diamond, version 2.0.13) was used for non-redundant gene set comparison against amino acid sequences in the NR database and taxonomic species annotation from the corresponding NR library database information. The abundance of each species was calculated by summing up the abundances of its corresponding genes. Unigenes were compared against functional databases, such as The Kyoto encyclopedia of genes and genomes (KEGG) and The Comprehensive Antibiotic Resistance Database (CARD), using DIAMOND software for functional annotation of genes at different levels, followed by calculation of relative abundance at functional and classification levels. Subsequently, various statistical analyses, including similarity clustering, grouping sorting, difference comparison, and visual display of the results, were performed.

### Statistical analysis

GraphPad Prism software (version 8) and the Majorbio platform were used for data analysis and graphing. Data are expressed as the mean ± standard deviation. One-way analysis of variance was used to compare the means between groups, and Tukey’s and Dunnett’s tests were used to compare the means between two groups. Differences were considered statistically significant when *P* < 0.05.

## Results

### JWC alleviated gastric mucosal inflammation and modulated inflammatory cytokines

**Figure 2:**
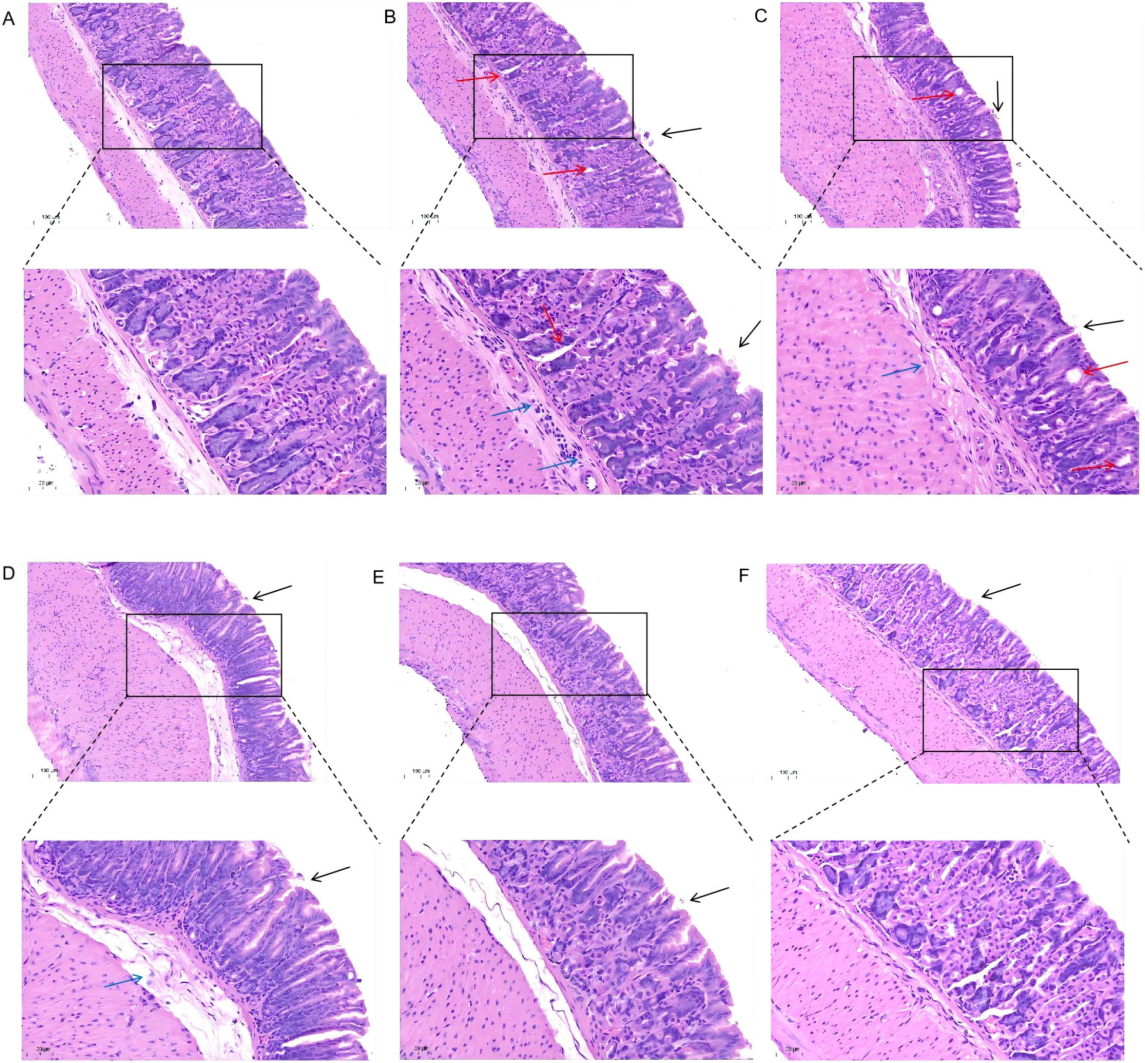
H&E staining to observe the effect of JWC on gastric mucosal damage in mice. **(A)** Control group. **(B)** Model group. **(C)** AC triple group. **(D)** JWCL group. **(E)** JWCM group. **(F)** JWCH group. epithelial cell exfoliation (black arrows), mild dilation of gastric glands (red arrows), and inflammatory cell infiltration (blue arrows). (20× magnification, scale bar 100 µm; 40× magnification, scale bar 50 µm).

Following *H. pylori* infection, mice in the Model group exhibited slower weight gain compared to the Control group (**Figure 1b**). Successful colonization of *H. pylori* was confirmed by a positive RUT (**Figure 1c**) and visualization of bacteria via WS silver stain (**Figure 1d**). Histopathological examination revealed that *H. pylori* infection induced damage to the gastric mucosal epithelium and inflammatory cell infiltration. These pathological changes were markedly alleviated in the JWCM and JWCH groups, which showed relatively intact mucosal structures similar to the Control group (**Figure 2**).

**Figure 3:**
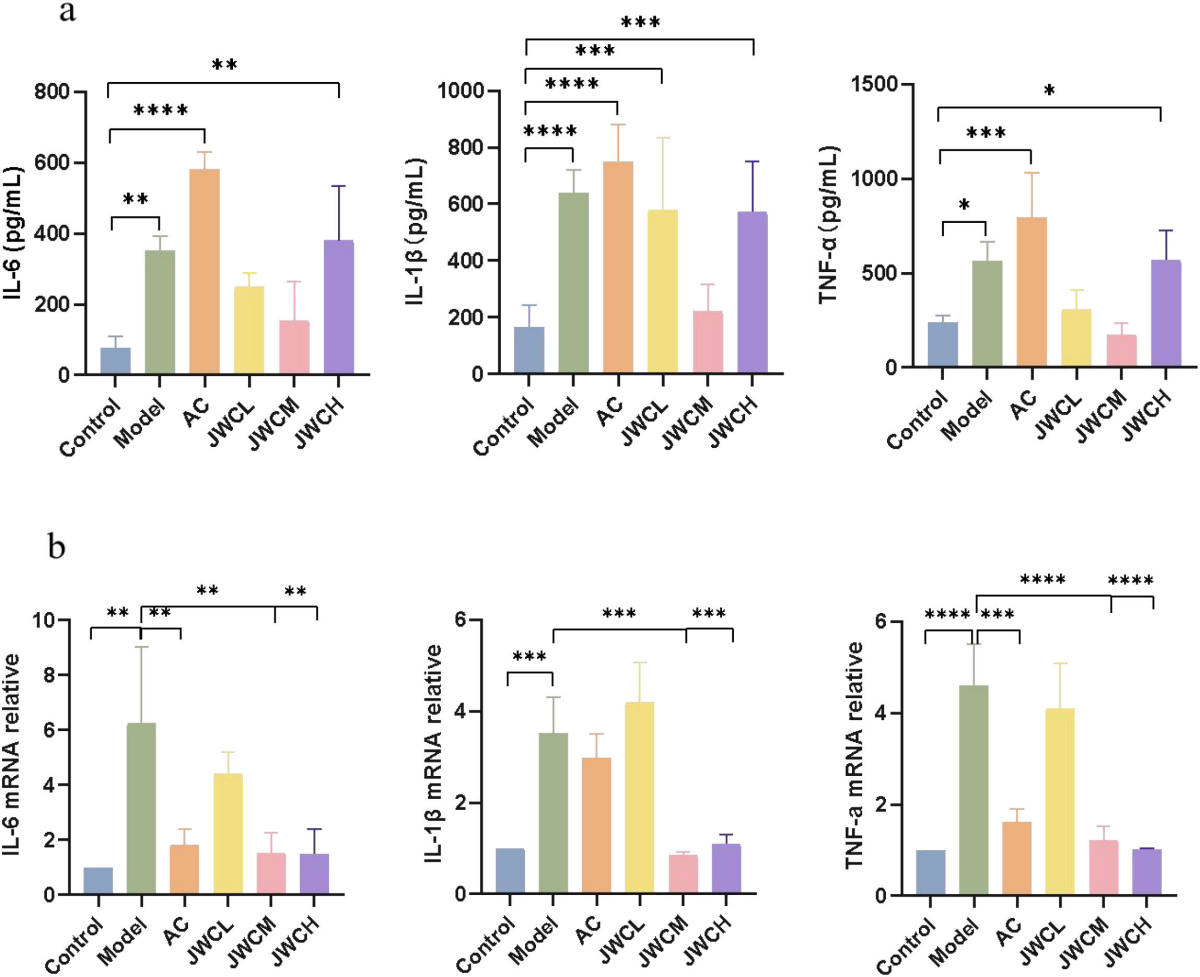
JWC regulated the expression of inflammation factors in gastric sinus tissues of (*H*) *pylori* infected mice. **(a)** The contents of IL-6, IL-1β and TNF-α. **(b)** The mRNA expression level of IL-6, IL-1β and TNF -α. The results of each experiment are the mean ± standard deviation of three independent experiments, *P < 0.05, **P < 0.01, ***P < 0.001, ****P < 0.0001.

Consistent with the histopathological findings, *H. pylori* infection significantly increased the levels of pro-inflammatory cytokines IL-6, IL-1β, and TNF-α in gastric antral tissue supernatants. While the AC triple therapy group showed a continued increase in these cytokines, JWC treatment, particularly at medium and high doses, significantly downregulated their concentrations (**Figure 3a**). Similarly, qRT-PCR analysis confirmed that JWC treatment significantly reduced the mRNA expression levels of IL-6, IL-1β, and TNF-α in the gastric mucosa (**Figure 3b**).

### Analysis of gut microbial diversity and composition

**Figure 4:**
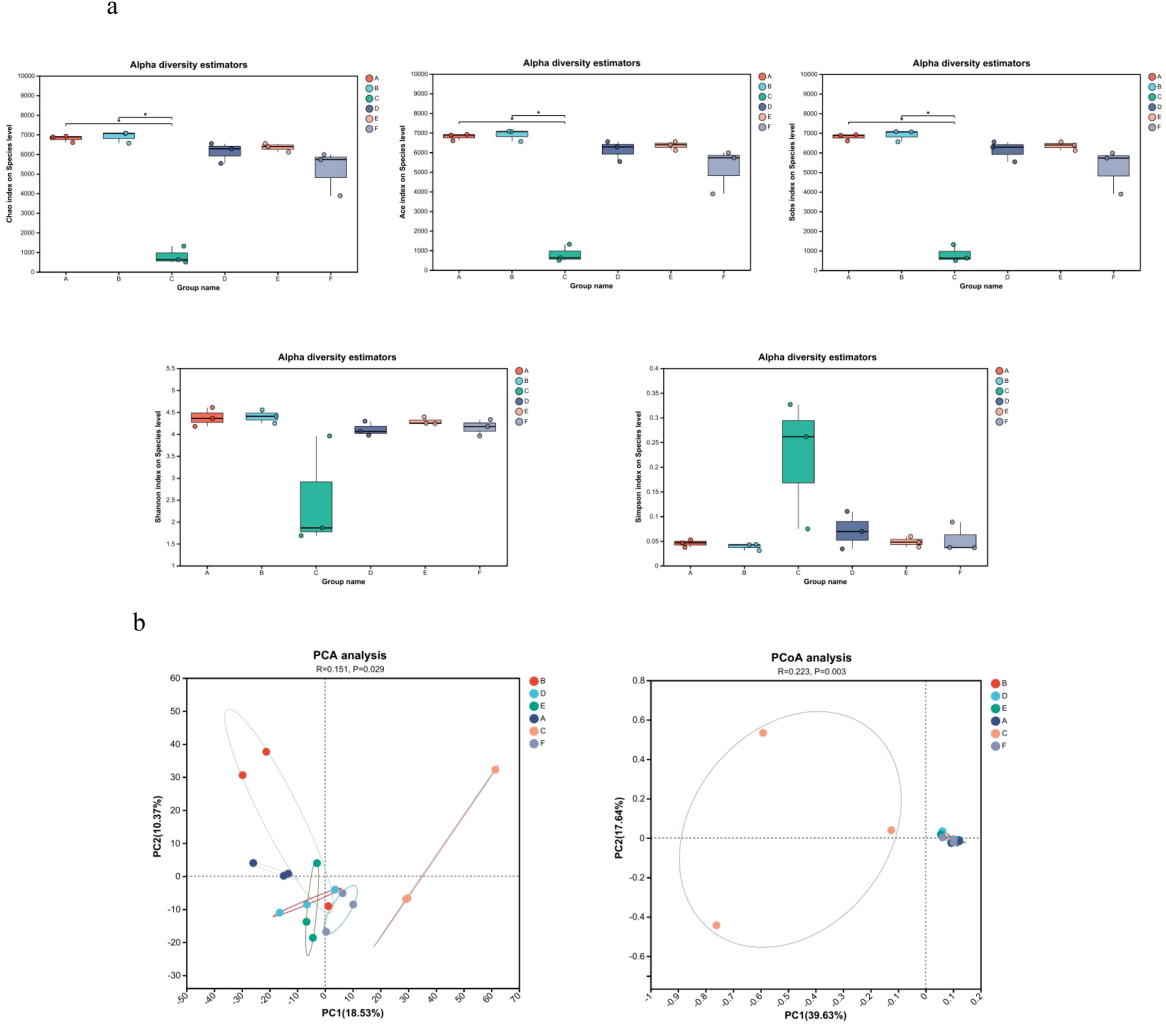
Microbial diversity analysis. **(a)** Microbial α diversity analysis. Chao index difference analysis, Ace index difference analysis, Sobs index difference analysis, Shannon index difference analysis and Simpson index difference analysis. **(b)** Microbial β diversity analysis. PCA analysis and PCoA analysis. A, Control group; B, Model group; C, AC triple group; D, JWCL group; E, JWCM group; F, JWCH group. *P < 0.05.

**Figure 5:**
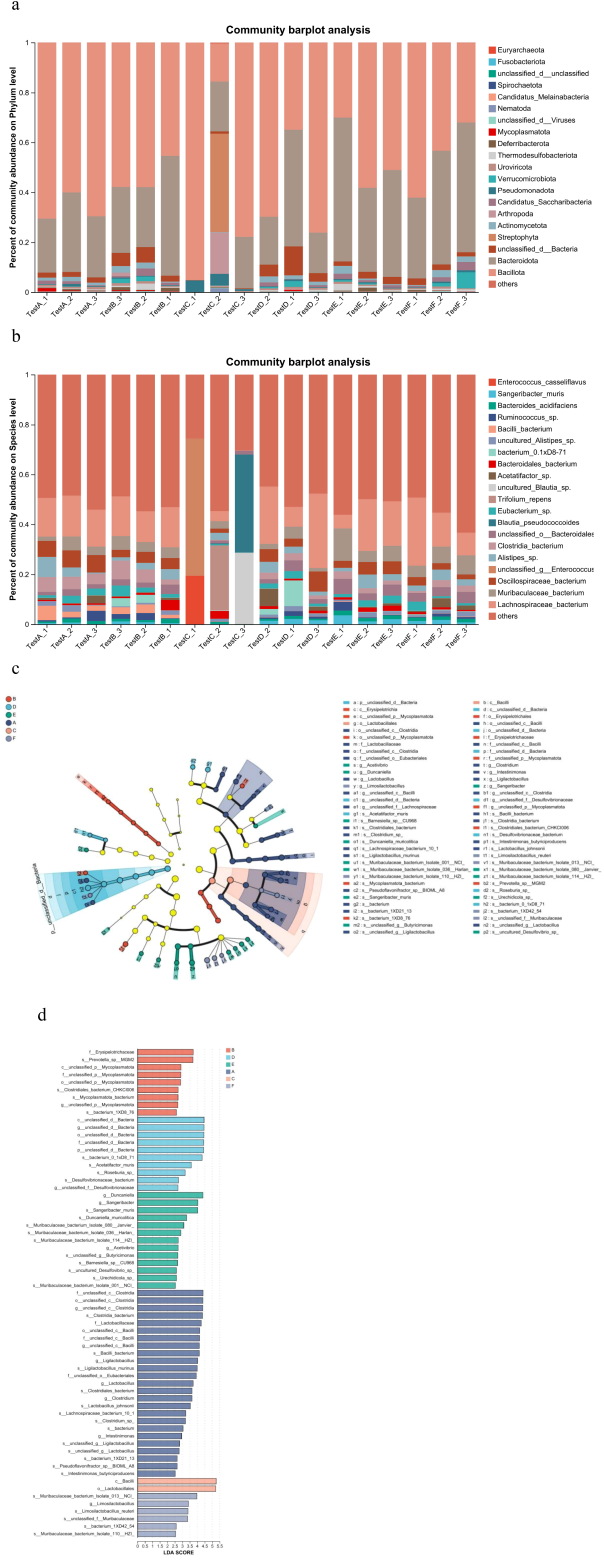
Analysis of microbial composition and difference. **(a)** Relative abundance of microorganisms at the phylum level. X-axis: Sample names. The labels testA-1, testA-2, and testA-3 correspond to the individual samples in Group (A) Y-axis: Relative abundance of species. Each colored segment in the bar plot represents a different species, and its length denotes the proportion of that species in the sample. **(b)** Relative abundance of microorganisms at the species level. X-axis: Sample names. Y-axis: Relative abundance of species. Each colored segment in the bar plot represents a different species, and its length denotes the proportion of that species in the sample. **(c)** LEFSe multi-species hierarchical tree. Statistical analyses were performed only from the phylum to the species level. Nodes of different colors represented microbiomes significantly enriched in the corresponding group. The diameter of each circle was proportional to the abundance of the group. Yellow nodes indicated microbiomes that were not significantly different between groups. **(d)** LDA discrimination result map (LDA score>2.5). A, Control group; B, Model group; C, AC triple group; D, JWCL group; E, JWCM group; F, JWCH group.

Analysis of α diversity, reflecting species richness and evenness, showed no significant differences between the Model and Control groups (**Figure 4a**). In contrast, the AC triple therapy group exhibited significantly reduced Chao, Ace, Sobs, and Shannon indices, alongside an elevated Simpson index, indicating markedly decreased microbial diversity. β diversity analysis, assessing inter-group compositional differences, revealed substantial separation of the AC triple group from others in PCA/PCoA plots, while JWC-treated groups clustered closely with Control group, suggesting minimal microbial community disruption (**Figure 4b**). Taxonomic analysis demonstrated pronounced dysbiosis in AC triple group. At the phylum level (**Figure 5a**), AC treatment led to drastic reduction in Bacteroidota (7% vs 14%) and Actinomycetota (1% vs 16%), with abnormal expansion of Streptophyta (99% vs 0), Pseudomonadota (61% vs 9%) and Arthropoda (92% vs 0). Species level analysis (**Figure 5b**) showed near disappearance of *Lachnospiraceae_bacterium* (2% vs 18%), *Oscillospiraceae_bacterium* (3% vs 28%), *Muribaculaceae_bacterium* (5% vs 14%), and *Clostridia_bacterium* (1% vs 31%), while opportunistic pathogens like *Enterococcus* and *Blautia pseudococcoides* proliferated extensively. The JWCM group exhibited a microbial profile characterized by an increased abundance of Bacteroidota (24% vs 14%) at the phylum level, while maintaining levels of Actinomycetota, Streptophyta, and Arthropoda comparable to Control group. Notably, *Muribaculaceae_bacterium* showed substantial enrichment (30% vs 14%), whereas *Oscillospiraceae_bacterium* (18% vs 28%) and *Clostridia_bacterium* (13% vs 31%) were reduced. The abundances of *Lachnospiraceae_bacterium* and *Alistipes_sp* remained similar to Control group.

Community structure analysis revealed group-specific microbial signatures through hierarchical clustering (**Figure 5c**) and LDA effect size discrimination (**Figure 5d**). Significant biomarker taxa were identified for each group: *g:unclassified_c:Clostridia* and *f:Lactobacillaceae* in Control group; *g:unclassified_p:Mycoplasmatota* in Model group; *c:Bacilli* and *o:Lactobacillales* in AC triple group; and *Muribaculaceae_bacterium* in JWC-treated groups.

### Analysis of antibiotic resistance genes

**Figure 6:**
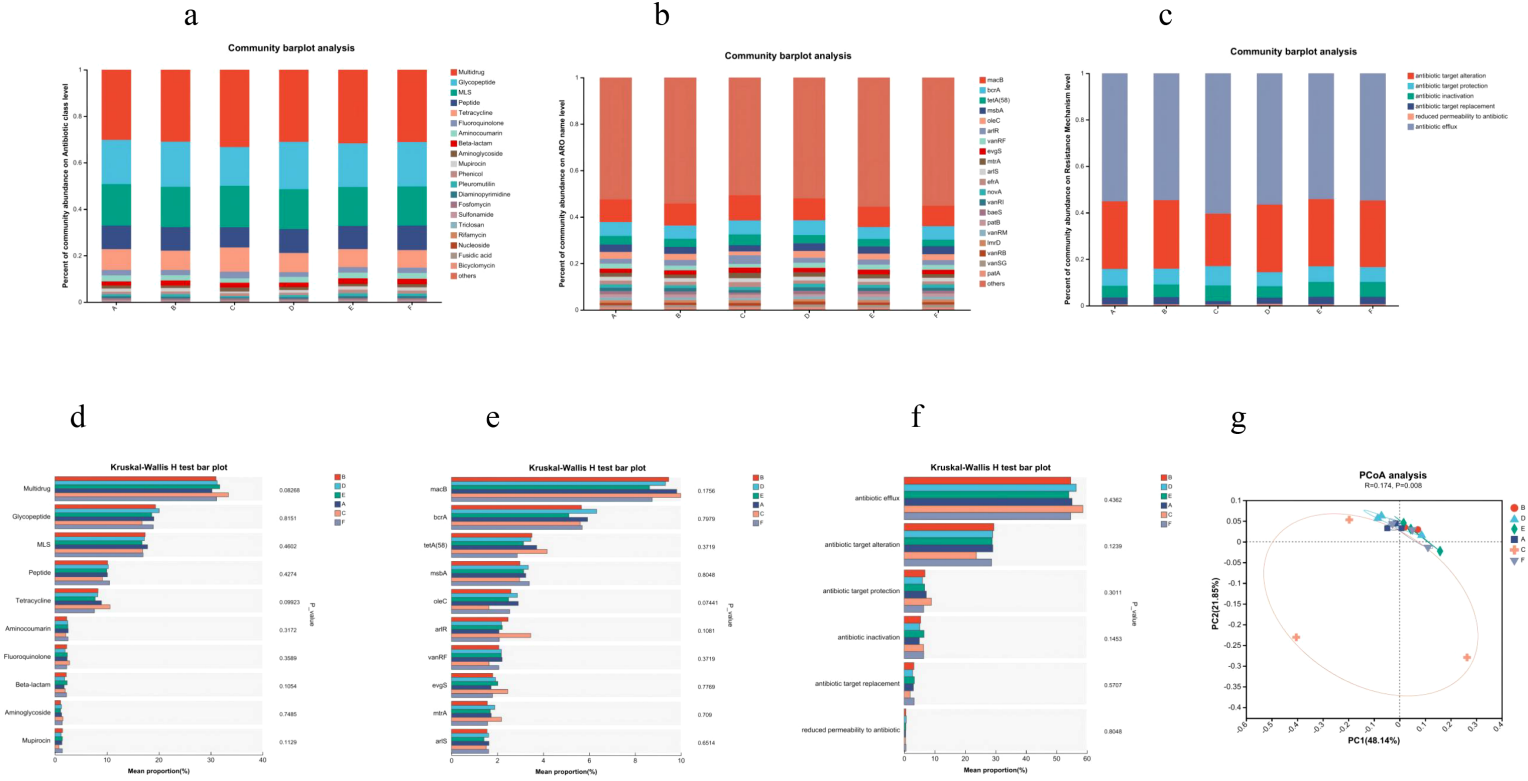
**CARD results. (a)** Distribution of antibiotic classes in the top 20 of abundance. **(b)** Distribution of the top 20 AROs in abundance. **(c)** Distribution of major resistance mechanisms. **(d)** Antibiotic class analysis of variance. **(e)** ARO Difference analysis. **(f)** Resistance mechanism difference analysis. **(g)** PCoA analysis. A, Control group; B, Model group; C, AC triple group; D, JWCL group; E, JWCM group; F, JWCH group.

Principal coordinate analysis revealed significant separation of the AC triple therapy group from other groups in the resistome profile (**Figure 6g**). The AC triple group showed enrichment in antibiotic resistance genes (ARGs) across multiple classes, including multidrug (18% vs 16%), tetracycline (20% vs 17%), fluoroquinolone (20% vs 17%), and aminoglycoside (23% vs 16%) resistance. Specifically, key ARGs such as *macB* (19% vs 17%), *arlR* (25% vs 14%), *evgS* (19% vs 15%), *tetA(58)* (22% vs 17%), and *mtrA* (22% vs 16%) were elevated (**Figures 6a, b, d, e**). Mechanistically, AC therapy increased genes encoding efflux pumps (18% vs 16%), antibiotic target protection (20% vs 17%), and antibiotic inactivation (19% vs 14%), while reducing those involved in target alteration (13% vs 17%) and replacement (9% vs 18%) (**Figures 6c, f**). In contrast, JWCM treatment downregulated tetracycline (15% vs 17%) and aminoglycoside (14% vs 16%) resistance levels, with specific suppression of *macB*, *tetA(58*), *bcrA*, *oleC*, and *arlS* genes. The overall ARG profile in JWCM group resembled that of Control group, demonstrating JWC’s capacity to restrict the expansion of the gut resistome.

### KEGG pathway enrichment and MAPK pathway activation

**Figure 7:**
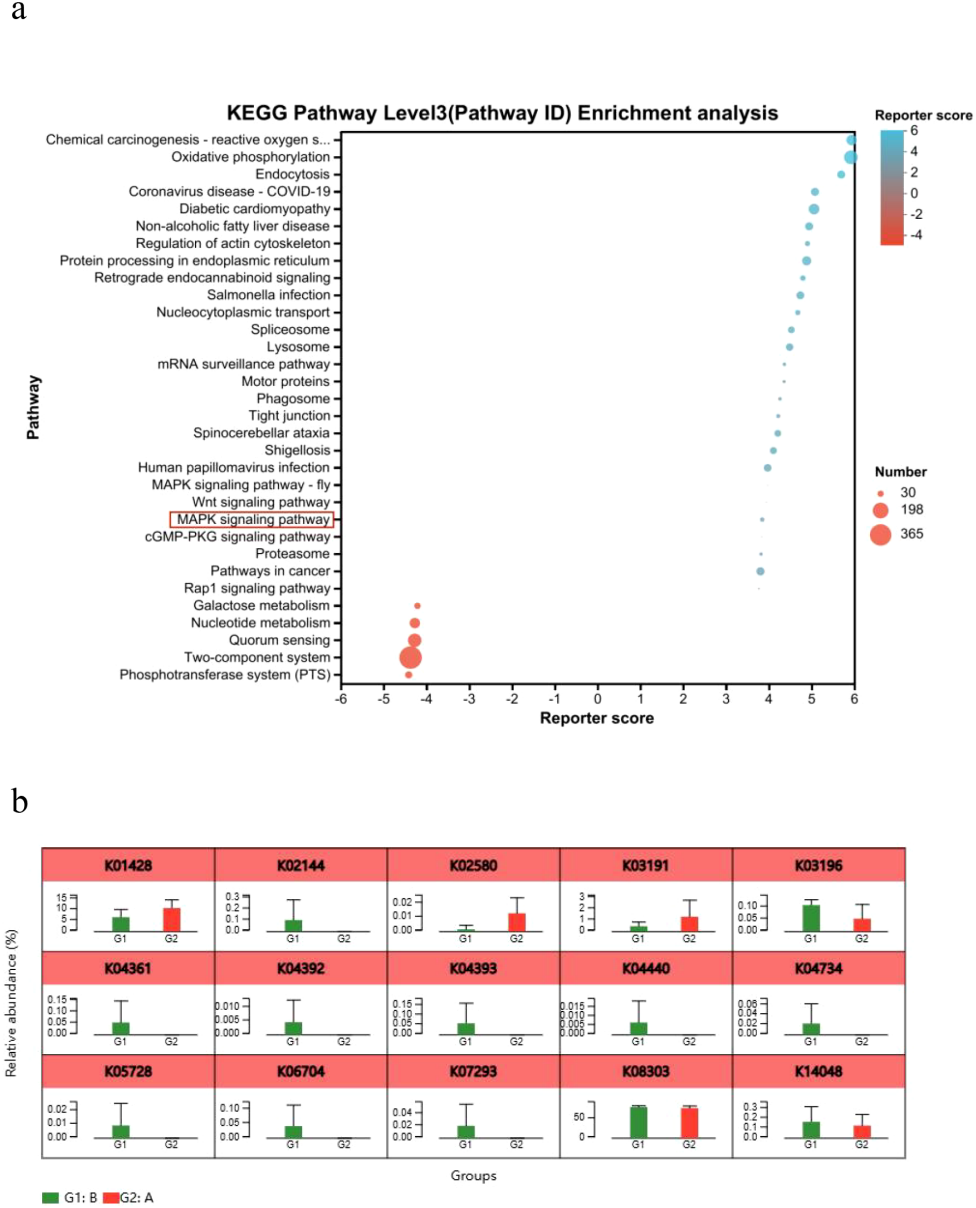
KEGG pathway enrichment analysis. **(a)** Bubble plot of KEGG functional enrichment analysis. **(b)** Analysis of relative gene abundance of key enzymes in the epithelial cell signaling pathway in *H*. *pylori* infection. A, Control group; B, Model group; *P < 0.05, **P < 0.01.

KEGG analysis revealed significant enrichment of the MAPK signaling pathway in the Model group compared to Controls (**Figure 7a**). Consistent with this, key genes (K04361, K06704, K07293, K05728) involved in epithelial cell signaling during *H. pylori* infection were upregulated (**Figure 7b**), confirming MAPK pathway activation.

**Figure 8:**
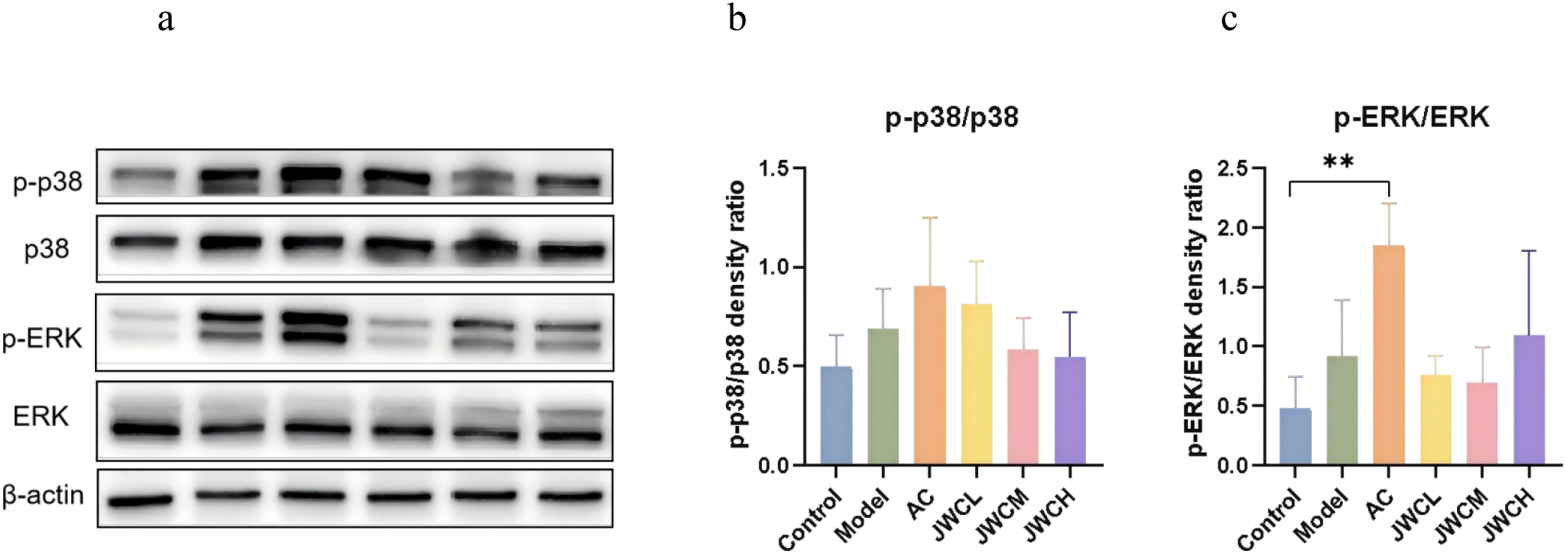
JWC regulated the expressions of MAPK pathway proteins in (*H*) *pylori*-infected mice. **(a)** WB detection of p38, p-p38, ERK, p-ERK expression. **(b)** Relative expression of p-p38/p38. **(c)** Relative expression of p-ERK/ERK. Results of each experiment are the mean ± standard deviation of three independent experiments, *P < 0.05, **P < 0.01.

At the protein level, phosphorylation of ERK and p38 was elevated in infected mice. While AC triple therapy sustained this activation, JWC treatment significantly suppressed both p-ERK and p-p38 expression (**Figure 8**), indicating direct modulation of the MAPK pathway.

## Discussion

*H. pylori* establishes persistent colonization in the gastric epithelium, leading to chronic inflammation that can progress to peptic ulcers and gastric cancer (Kumar et al., 2021). This study demonstrates that JWC effectively alleviates *H. pylori*-induced gastric inflammation in mice. Its mechanism appears to be multi-faceted, involving the preservation of intestinal microbial ecology, mitigation of antibiotic resistance gene enrichment, and modulation of the host MAPK signaling pathway, offering a contrasting profile to the ecological drawbacks of conventional triple therapy.

Compared to the Control group, mice in the Model group exhibited a slower increase in body weight following intraperitoneal injection of cyclophosphamide and *H. pylori*. On days 7 and 14 of modeling, the body weights of mice in the Model group were lower than that of the control group, indicating that *H. pylori* infection can impede mouse growth rate (**Figure 1b**). A meta-analysis demonstrated that eradication of *H. pylori* is associated with an increase in body weight and mass index (Upala et al., 2017). The body weight of *H. pylori*-infected mice was also lower in animal experiments (Noh et al., 2017), potentially indicating an association between *H. pylori* and alterations in body metabolism.

Our findings confirm that JWC significantly mitigates gastric mucosal damage and reduces the levels of key pro-inflammatory cytokines, including IL-6, IL-1β, and TNF-α. A notable observation was that while the AC triple therapy effectively eradicated the pathogen, it failed to significantly reduce IL-1β levels. This phenomenon may be attributed to several factors. First, antibiotics can induce gastrointestinal discomfort and transient mucosal stress, potentially perpetuating local inflammatory cytokine release even during pathogen clearance (Zhao et al., 2021). Second, as our metagenomic sequencing revealed, AC therapy caused severe gut dysbiosis, characterized by a loss of diversity and an expansion of opportunistic pathogens like *Enterococcus*. Such dysbiosis can adversely modulate the host immune system, potentially sustaining the production of pro-inflammatory cytokines (Ye et al., 2020; Nabavi-Rad et al., 2022). Third, and perhaps most critically, the AC regimen did not significantly inhibit the activation of the MAPK signaling pathway, a key regulator of IL-1β synthesis. In contrast, JWC treatment effectively suppressed this pathway, providing a plausible mechanism for its superior control of this specific cytokine.

Consistent with previous reports (Iino et al., 2020; Cui et al., 2022), *H. pylori* was undetectable in the intestinal microbiota of infected mice, indicating a lack of bacterial translocation from the stomach. This supports the notion that *H. pylori* influences the gut ecosystem indirectly—likely through host immune modulation or gastric environmental changes (Chen et al., 2021). While some studies report variable effects of *H. pylori* on gut microbial diversity (Kienesberger et al., 2016; Chen et al., 2021), we observed no significant alteration in α- or β-diversity in KM mice, possibly due to the short infection duration and low gastric colonization density observed histologically. In contrast, AC triple therapy markedly reduced both α diversity and β diversity, aligning with known antibiotic-induced microbial depletion (Du et al., 2024). Notably, JWC treatment preserved intestinal microbial structure and composition, showing no significant divergence from the Control and Model groups. This suggests that JWC exerts minimal disruptive effects on the gut microbiota while alleviating gastric inflammation.

The focus on intestinal microbiota rather than gastric microbiota was deliberate, as the gut serves as a primary site for antibiotic resistance development and systemic immune regulation. Furthermore, the low bacterial biomass of the murine stomach poses technical challenges for metagenomic analysis. Gastric infection and inflammation were robustly assessed via histopathology and cytokine profiling. Future study incorporating concurrent gastric microbial analysis would provide a more comprehensive perspective (Wang C. et al., 2023).

*Muribaculaceae* and *Alistipes_sp*, belonging to Bacteroidota, metabolize propionic acid. *Oscillospiraceae_bacterium*, *Lachnospiraceae_bacterium*, and *Clostridia_bacterium* are primary butyric acid producers. These short-chain fatty acids (SCFAs) serve as the main energy source for colonic epithelial cells and help lower intestinal pH, inhibiting pathogens, promoting probiotics, and enhancing nutrient absorption (Agus et al., 2021). SCFAs also protect the intestinal mucosal barrier and exhibit anti-inflammatory, anti-tumor, and immune-regulatory functions (Dalile et al., 2019). In contrast, *Enterococcus*, an opportunistic pathogen tolerant to salt and acid, often shows β-lactam resistance due to its weak-binding penicillin-binding proteins (Torres et al., 2018). A meta-analysis revealed that in the short-term follow-up period post-eradication, the abundance of *Enterobacteriaceae* and *Enterococcus* at the genus level increased (Ye et al., 2020). The AC triple therapy profoundly diminished gut microbiota diversity and richness, drastically altering its composition by depleting beneficial SCFAs and promoting opportunistic pathogens. In contrast, JWC treatment preserved microbial diversity and promoted a beneficial compositional shift, notably increasing the abundance of *Muribaculaceae*, a bacterium associated with anti-inflammatory properties. Recent evidence suggests that dietary components profoundly influence gut microbial composition and function (Liu et al., 2023; Yang et al., 2025). The plant-derived ingredients in JWC may mimic dietary fibers or polyphenols, thereby promoting the growth of SCFA-producing bacteria and helping to counteract antibiotic-induced dysbiosis by supporting a resilient microbial network. While we did not directly measure SCFAs levels, the observed structural changes are consistent with a shift towards a more favorable microbial state.

A significant increase in antibiotic resistance genes was observed following the completion of bismuth quadruple therapy for *H. pylori* eradication, consisting of amoxicillin, clarithromycin, bismuth, and esomeprazole (Abdulkhakov et al., 2024). The genes were primarily associated with resistance to β-lactam antibiotics, aminoglycosides, fluoroquinolones, macrolides, and glycopeptides. Concomitant with its impact on microbiota composition, AC therapy elevated the abundance of a broad spectrum of antibiotic resistance genes in the gut resistome in our study. This enrichment suggests that antibiotic pressure can selectively favor resistant commensals, potentially augmenting the reservoir for horizontal gene transfer (Malfertheiner et al., 2017; Liou et al., 2023). However, JWC downregulated these resistance genes. It is critical to distinguish this gut resistome from pathogen-specific resistance. Our study didn’t characterize the primary resistance mechanisms within the *H. pylori* strains themselves, such as clarithromycin resistance *(23S rRNA* mutations) or metronidazole resistance (*rdxA* mutations), which are key drivers of clinical eradication failure (Iqbal et al., 2024). Therefore, elucidating the comprehensive impact of JWC on antimicrobial resistance represents a critical future research direction.

*H. pylori* infection activates the epidermal growth factor receptor (EGFR) pathway, initiating downstream ERK signaling (Du et al., 2007). This process involves the upregulation of epidermal growth factor (EGF) and ADAM metallopeptidase domain 10 (ADAM10), which disrupts the shedding of heparin-binding EGF (HB-EGF), leading to EGFR transactivation (Keates et al., 2001). Furthermore, the bacterial cytotoxin-associated gene A (CagA) protein is translocated into host cells via the type IV secretion system, where it binds to and enhances the phosphatase activity of SHP2 in a phosphorylation-dependent manner, thereby activating both RAS-dependent and independent MAPK cascades (Maroun et al., 2000; Schaeper et al., 2000). Although transcriptional upregulation of EGFR, ADAM10, and SHP2 genes in the Model group was not statistically significant, metagenomic analysis confirmed significant enrichment of the MAPK pathway, accompanied by increased phosphorylation of ERK and p38. This indicates that *H. pylori* infection triggers a cascade within the MAPK signaling pathway. This indicates that *H. pylori* infection triggers a cascade within the MAPK signaling pathway, which is consistent with previous reports (Keates et al., 1999; Slomiany and Slomiany, 2001; Slomiany and Slomiany, 2002).

The MAPK pathway, comprising ERKs, JNKs and p38, is crucial for regulating cell proliferation, differentiation, and inflammatory responses (Morrison, 2012). Our findings demonstrate that JWC treatment significantly inhibits the phosphorylation of p38 and ERK. This suppression of MAPK activation likely represents a key mechanism by which JWC mitigates gastric mucosal inflammation. In contrast, AC triple therapy showed no significant effect on this pathway. The anti-inflammatory effects of JWC appear to operate primarily through these host-directed pathways rather than solely via direct bactericidal activity. It should be noted that this study did not evaluate the direct antibacterial effect of JWC. Future research should quantify the gastric *H. pylori* load through CFU counts to provide a more direct and precise measure of JWC’s antibacterial efficacy.

Beyond therapeutic development, the field of *H. pylori* management is also advancing in diagnostics. Techniques such as Linked Color Imaging and Confocal Laser Endomicroscopy now enable precise detection and real-time, cellular-level visualization of *H. pylori* gastritis, with some methods allowing objective quantification of mucosal inflammation (Sun et al., 2023). Although these tools remain limited by cost and technical demands, they offer promising avenues for translational research. Future clinical studies on JWC could leverage such non-invasive imaging to quantitatively monitor gastritis resolution, providing a synergistic approach to treatment evaluation.

## Conclusion

In contrast to AC triple therapy which induces gut dysbiosis and expands the antibiotic resistome, JWC effectively ameliorates gastric inflammation while preserving intestinal microbial diversity and richness, reducing the abundance of antibiotic resistance genes, and suppressing MAPK signaling pathway activation.

We acknowledge the limitations of our current study. Future study should: first, quantitatively evaluate JWC’s direct antibacterial efficacy through CFU counting to strengthen the evidence of its antimicrobial activity; second, perform functional analysis of microbial metabolites, particularly SCFAs, to validate the physiological relevance of the observed microbiota shifts; and third, identify the precise molecular targets of JWC within the MAPK pathway to elucidate its mechanism of action.

## Data Availability

The datasets presented in this study can be found in online repositories. The names of the repository/repositories and accession number(s) can be found in the article/**Supplementary Material**.
