# Supplementary material for: Jinghuaweikang capsule alleviates Helicobacter pylori-infected gastric mucosal inflammation and drug resistance by regulating intestinal microbiota and MAPK pathway
Source: Front Cell Infect Microbiol. 2025 Nov 28;15:1628594. doi: 10.3389/fcimb.2025.1628594 (PMC12698565; doi:10.3389/fcimb.2025.1628594)
Supplement: Supplementary file 1 [file DataSheet1.pdf]

Supplementary Material

Chromatographic Conditions

Separation was performed using an Agilent DB-17 capillary column (30 m × 0.32 mm, 0.25 μm film thickness; stationary phase: 50% phenyl-methyl polysiloxane). The temperature program was set as follows: initial temperature 60°C, increased to 80°C at a rate of 1.5 °C·min^{-1}, then raised to 200°C at a rate of 60°C·min^{-1}. The injector temperature was maintained at 250°C. Detection was carried out using a Flame Ionization Detector (FID) operated at 250°C. High-purity nitrogen served as the carrier gas. Samples were injected in split mode with a split ratio of 15:1, and the injection volume was 1 μL.

Preparation of Solutions

The reference solution was prepared by accurately weighing an appropriate amount of p-cymene reference standard and dissolving it in ethyl acetate to obtain a solution with a concentration of 0.5 mg·mL^{-1}.

The test solution was prepared by accurately weighing approximately 60 mg of the contents of JWC into a 10 mL volumetric flask. The sample was dissolved in and made up to volume with ethyl acetate.

Determination of the Characteristic Peaks in the Fingerprint

The GC data from twenty batches of JWC were processed using the "Similarity Evaluation System for Chromatographic Fingerprint of Traditional Chinese Medicine (2004A)" to establish the characteristic fingerprint and calculate similarity. The results demonstrated that the similarities between all batch-specific fingerprints and the computer-generated reference fingerprint were greater than 0.90. Analysis via the software identified four common peaks in the GC fingerprints as the characteristic markers for this preparation. The development of a characteristic fingerprint requires the selection of a suitable reference substance that exhibits satisfactory peak shape, good resolution, stable retention time, and represents a major pharmacologically active component. In this study, p-cymene—the primary constituent of the monarch herb *Chenopodium ambrosioides* L. in JWC—was selected as the reference standard. Peak 2 was unambiguously identified as p-cymene.

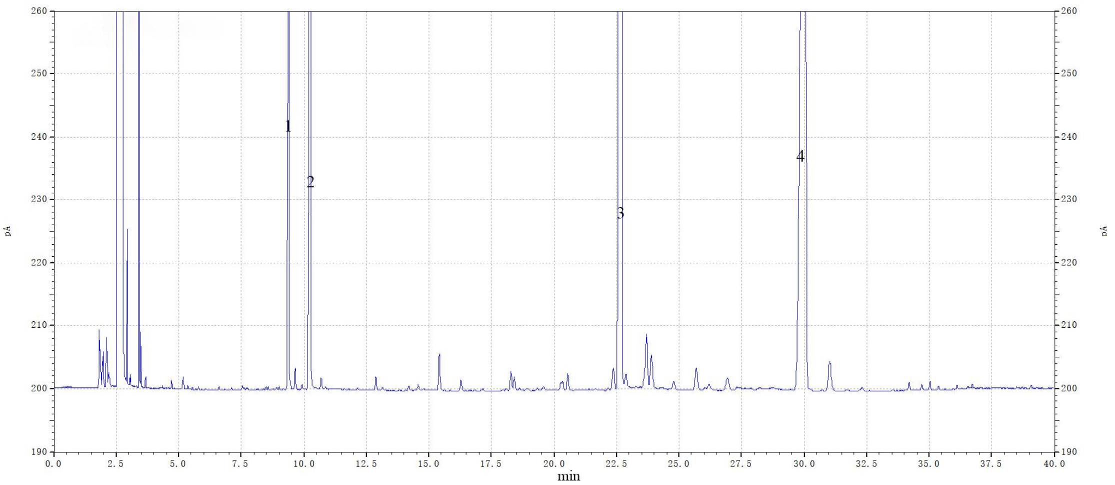

Figure S1 GC Fingerprint Chromatogram of JWC. Peak 1: α-Terpinene; Peak 2: p-Cymene
